# China’s dam-builders: their role in transboundary river management in South-East Asia

**DOI:** 10.1080/07900627.2017.1329138

**Published:** 2017-06-13

**Authors:** Frauke Urban, Giuseppina Siciliano, Johan Nordensvard

**Affiliations:** a Center for Development, Environment and Policy (CeDEP), SOAS, University of London, London, UK; b Department of Sociology, Social Policy and Criminology, University of Southampton, Southampton, UK

**Keywords:** Hydropower, China’s dam-builders, South-East Asia, Mekong, transboundary river management

## Abstract

This article investigates China’s role as the world’s largest builder of and investor in large dams, focussing on the Greater Mekong Sub-Region in South-East Asia. It addresses the role Chinese actors play in dam-building as well as the environmental, social, economic and political implications by drawing on case studies from Cambodia and Vietnam. The article finds that China’s dam-building is perceived very differently in different countries of South-East Asia. In Cambodia, the dams in the Greater Mekong Sub-Region are considered instruments of economic growth and development, whereas downstream in Vietnam the dams are seen as potentially undermining national growth, development and security.

## Introduction

Large dams have been the subject of controversy and debate for several decades as a result of their far-reaching and often irreversible social and environmental impacts (World Commission on Dams, 2000). In the pursuit of renewable, low-carbon energy and climate change mitigation, hydropower is experiencing a renaissance in many parts of the world, despite its vulnerability to climate change (Intergovernmental Panel on Climate Change, 2011). At the forefront of the renaissance of large hydropower dams is China, the world’s largest dam-builder. Internationally, China’s engagement in the hydropower sector is reported to be primarily through Chinese state-owned enterprises (SOEs) such as Sinohydro (also known as PowerChina or PowerChina Resources Limited), a firm leading the global hydropower sector in terms of number and size of dams built, investment amounts, and global coverage.

While China has a long history of domestic dam-building, recent developments have led to Chinese dam-building overseas, particularly in low- and middle-income countries (LMICs) in Asia and Africa (Bosshard, 2009; International Rivers, 2012; McDonald, Bosshard, & Brewer, 2009). In recent years, Chinese dam-builders and investors have been particularly active in neighbouring countries along transboundary rivers, such as in the Mekong Region, with increased Chinese investments and dam-building especially in Myanmar, Laos and Cambodia.

China’s rapid economic growth means that the large-dams sector is nearly saturated in China; many big rivers have already been dammed, so the domestic market is very limited. The Chinese government’s Going Out strategy (Mohan & Power, 2008) and its recent One Belt, One Road policy (Xinhua Finance Agency, 2015) therefore encourage overseas investments to access overseas markets for Chinese goods and services, especially infrastructure, and partly also to access natural resources such as minerals, water and energy (Mohan & Power, 2008). It is estimated that there are currently close to 350 Chinese-funded and Chinese-built overseas hydropower projects listed as either completed, under construction, at the memorandum-of-understanding stage or suspended, most of them in South-East Asia (38%) or Africa (26%) (International Rivers, 2016). These data have to be treated with caution, though, as not all of these dams will necessarily be constructed. In South-East Asia, about 30 dams were completed by 2016 where Chinese dam-builders or financiers were involved, out of more than 120 dams that were proposed, under construction, suspended or completed (International Rivers, 2016). Data limitations exist, however, as the NGO International Rivers is currently the only organization tracking Chinese-built and Chinese-funded dams at the global level, while other databases are regional and hence incomplete.

The great majority of these dams are large, with capacity over 50 MW. Many have been built since 2000, in a time when other dam-building nations and organizations, particularly those from the OECD, have scaled down their operations.

As China has internationalized its dam-building industry, there have been questions over the motives and mechanisms of Chinese banks and SOEs. Chinese dam-builders are seen as different and distinct actors for a range or reasons, forming part of a wider logic of ‘encompassing accumulation’ that combines state-capital agendas as opposed to strictly profit-oriented (Lee, 2014). Some argue that their bundling of aid, trade and investments is a unique financing model. For example, SOEs like Sinohydro benefit from abundant state funding, especially through preferential loans from the ExIm Bank of China. Finally, the Chinese banks’ and firms’ pragmatic approach to regional politics and political alliances and associated tendency not to apply conditionality in their lending may result in ignoring poor governance in recipient countries. Playing into this is a perception that China is ‘all-powerful’ and drives these agendas, though recent work has argued that the agency of the recipient states is key in shaping how and in whose interests these investments play out (Carmody & Kragelund, 2016; Mohan & Lampert, 2013; Urban, Siciliano, et al., 2015). Yet, there may be other reasons: Kirchherr, Charles, and Walton (2016), and Kirchherr, Disselhoff, and Charles (2016) suggest that Chinese dam-builders are far cheaper and more effective in constructing dams than their competitors, with far fewer cost and construction-time overruns. Hensengerth (2013) found that the role of Chinese dam-builders is often strongly influenced by local host government settings, that their social and environmental performance is improving, and that essentially, Chinese dam-builders do not differ that much from their competitors.

At the same time, Chinese dam-builders play a major role in China’s transboundary river management, most importantly along the Mekong River. The Mekong River has become the ‘battery of Asia’, and will increasingly generate hydro-electricity for the region. It has also become a target for Chinese dam-builders as well as dam-builders from Thailand, Vietnam and elsewhere. China’s neighbours along the Mekong River are in a difficult situation. On one side they welcome Chinese investments in the energy and water sectors and hope to gain prosperity from these investments, as in Cambodia and Laos, while others fear for their water security and the irreversible impacts of China’s dam-building activities upstream, as in Vietnam.

This article investigates China’s role as the world’s largest builder of and investor in large dams, focussing on the Greater Mekong Sub-Region in South-East Asia. It addresses the role Chinese actors play in large dam-building as well as the environmental, social, economic and political implications of this by drawing on two case studies in Cambodia and Vietnam.

The article consists of the following sections. The next section elaborates the conceptual framework and the methodology of the research. Afterwards the findings are presented; then the findings are discussed, and the article concludes with recommendations.

## Conceptual framework and methodology

### Conceptual framework: political ecology of the Asian Drivers

The impacts of large hydropower dams are well known (e.g. Adams, 2000; Biswas, 1982; Scudder, 2012; Tilt, Braun, & He, 2009; Tortajada, Altinbilek, & Biswas, 2012). There is also abundant research on large dams in China (e.g. Chang, Liu, & Zhou, 2009; Dore & Yu, 2004; Hayashi, Murakami, Xu, & Watanabe, 2008; Hwang, Xi, Cao, Feng, & Qiao, 2007; Magee, 2006). A few studies have looked at China and its hydropower role overseas (Bosshard, 2009; Goh, 2004; McDonald et al., 2009; Yu, 2003). Yet, empirical research on China as a large dam-builder overseas is still in its infancy; until a few years ago much of the research on China’s environmental and social impact overseas was theoretical or conceptual rather than based on empirical evidence (compare Urban, Mohan, & Cook, 2013; Urban, Nordensvärd, Khatri, & Wang, 2013). This is slowly changing, with high-quality empirical work by Hensengerth (2013, 2016), Kirchherr et al. (2016), Matthews and Motta (2013), Lamb and Dao (2015), Chen and Landry (2016), Urban, Siciliano, et al. (2015), Urban, Nordensvard, Siciliano, and Li, (2015), Siciliano, Urban, Kim, and Lonn (2015), Tan-Mullins, Mang, and Urban (forthcoming) and several others in the last few years. However, the transboundary perspective has largely been omitted so far. This article is therefore one of the few to provide empirical evidence for this important topic.

This research uses a conceptual framework we term Political Ecology of the Asian Drivers (Siciliano & Urban, 2017; Siciliano, Urban, Tan-Mullins, Lonn, & Kim, 2016), which combines the theories and approaches of political ecology with those of the Asian Drivers. The Political Ecology of the Asian Drivers framework is a hybrid approach that seeks to elucidate three broad research aims, analyzing the actors driving the internationalization of dam-building; the channels through which they engage; and the environmental, social, economic and political impacts on the ground. We use this framework to overcome some of the current gaps in knowledge and understanding in relation to China’s rise and its environmental implications. The majority of early work on China’s engagement with LMICs was speculative (Mohan, 2008), economic (Jacques, 2009), and Africa-focused (Alden, Large, & Soares de Oliveira, 2008; Brautigam, 2009). Crucially, these studies largely ignored the environmental consequences of China’s internationalization (Shinn, 2016). Political ecology focuses on environmental and ecological implications of power relations for natural resource management, and this approach is therefore useful for understanding China’s impacts on overseas dam-building and related water management. Understanding a complex set of international actors, interdependencies and ecological impacts necessitates a broad theoretical framework (Urban, Nordensvärd, et al.,^ 2013^; Urban, Siciliano, et al.,^ 2015^) such as political ecology of the Asian drivers.

We combine the distinctive approach of the Asian Drivers (Humphrey & Messner, 2006) and their impacts with aspects of the political ecology approach to address China’s role in overseas dam-building. The Asian Drivers framework was developed to help understand the rise of Asian ‘tiger economies’ such as Korea, Singapore, Taiwan and later China. Developed by Humphrey and Messner (2005, 2006), Schmitz (2006), and Kaplinsky and Messner (2008), the framework assesses China’s and other East Asian countries’ direct and indirect impacts as rising powers and their channels of interaction with LMICs. The Asian Drivers framework focussed on impacts of economic channels of interaction, such as trade, aid and investments, as well as non-economic channels such as global governance, individuals and the environment (Kaplinsky & Messner, 2008). It also assessed direct and indirect impacts as well as the competitive and complementary dimensions of these impacts for the Asian ‘tiger economies’. The competitive and complementary aspect responded mainly to issues around international economic competitiveness. The framework was aimed at assessing the impacts of these emerging economies and their impacts on other countries, including developed and developing countries. In each of these channels – aid, trade, investment, global governance, individuals (including migrants from China and other East Asian countries) and environment – there will be a mixture of complementary and competitive economic impacts and positive and negative impacts in relation to society and the environment (Kaplinsky & Messner 2008). Urban, Mohan et al. (2013), Urban, Nordensvärd et al. (2013), and Urban, Nordensvärd, Wang, Khatri, & Mohan (2011) advanced the Asian Drivers framework further by addressing motives, actors and beneficiaries in addition to impacts, to analyze in more depth how, why and with which impacts Chinese actors engage in LMICs. The authors of this article amended the channel of interaction called ‘individuals’ into ‘society’ as this describes more adequately the dynamics of interaction and who benefits or loses from large dam projects.

However, the Asian Drivers framework is somewhat static, and while it focuses on actors and motives, it does not explicitly address questions of power. Nor does it analyze political economy drivers and ecological processes as entwined. To address these issues we use ideas from political ecology (Greenberg & Park, 1994; Perreault, Bridge, & McCarthy 2015; Wolf, 1972). The concepts of political ecology are essentially about human–environment relations (Perreault et al., 2015). They help to analyze the bargaining between actors involved in dam construction as well as the conflicts caused by the varied forms of control over access to natural resources such as water, energy, land and forests (Blaikie, 1985; Bryant & Bailey, 1997; Peet & Watts, 2004). Power relations between different actors are at the heart of the political ecology framework (Tan-Mullins, 2007), and assessing the unequal power relations between actors allows us to explain the uneven distribution of access and control of environmental resources. Bryant and Bailey (1997) developed three fundamental assumptions in practicing political ecology in developing countries. First, costs and benefits associated with environmental change are distributed unequally. Second, this unequal distribution inevitably reinforces or reduces existing social and economic inequalities. Third, the unequal distribution of costs and benefits and the reinforcing or reducing of pre-existing inequalities have political implications in terms of the altered power relationships that result.

Power is related to the differential ability to control and/or access the economic benefits from resource exploitation (Bryant 1996, 1997; Dauvergne, 1994; Peluso, 1992). Crucially, we examine power across actors and scales in relation to natural resource access (Dittgen, 2015). As we noted, it is often assumed that ‘the Chinese’ hold the power, but the agency of host governments as well as wider social actors is important. In terms of SOEs entering LMIC markets, we focus on the brokerage between state elites on both the Chinese and LMIC sides (see Carmody, Hampwaye, & Sakala 2012), in which infrastructure deals are often negotiated behind closed doors and bypass domestic channels of accountability.

The Political Ecology of the Asian Drivers framework enables us to address the role and impacts of Chinese dam-builders in LMICs, assess issues of power, and deal with the environmental and social implications for global hydropower development. The article also focuses on the views and motives of Cambodian and Vietnamese actors towards Chinese hydropower investment and transboundary river management.

We therefore focus on the following channels of interaction in this combined conceptual approach: trade, investment, aid, innovation, politics (addressing issues of power), environment and society. We also analyze the motives, actors, beneficiaries and impacts from both the Chinese side and the LMIC side.

## Methodology

The empirical research was conducted between 2012 and 2016 with interviews and focus group discussions (FGDs) in Cambodia, China and to a limited extent Vietnam. Cambodia and Vietnam were selected because they both have seen an increase in Chinese investments in the dam sector, though with different dynamics. Cambodia was selected as it is one of the countries that Chinese actors heavily invest in, including in the hydropower sector. The political, economic and cultural relations between China and Cambodia are closely knit, and both countries welcome bilateral trade and investment opportunities. Vietnam was selected as a contrasting case, as Chinese investment is less heavy and more indirect, for example Chinese actors investing in parts of the value chain or in joint ventures, rather than large Chinese hydropower firms leading large dam projects as in Cambodia. The political, economic and cultural relations between China and Cambodia are also rather complicated and ambivalent, which in turn creates a different relationship in terms of trade and investments, including in the hydropower sector. The two countries are also located at different places along the Mekong River. Vietnam is very much downstream, close to the sea; Cambodia is also downstream, but upstream from Vietnam, so dams built in Cambodia will have an impact on Vietnam’s water issues. We started by conducting a multi-level stakeholder mapping (Schiffer & Hauck, 2010) to identify key stakeholders engaged in Chinese overseas hydropower projects and in China itself.

We held 62 interviews and 10 FGDs in China, Cambodia and Vietnam: 20 key informant interviews with institutional actors who were involved in or had expertise in dam construction; 18 interviews with Chinese dam-builders and financiers; 24 interviews with affected community members and their chiefs; and 10 FGDs with local community members. For the institutional interviews, we conducted semi-structured interviews with hydropower firms, policy makers, financiers, NGOs and academics. These actors were interviewed about their perceptions of the socio-economic and environmental impacts of dams, governance issues and political implications, as well as the role of Chinese dam-builders. On the Chinese side, semi-structured interviews were held with dam-builders and financiers such as Sinohydro, the Export-Import Bank of China, and government officials from authorities like the Ministry for Water Resources and the Ministry of Finance and Commerce. This enabled us to investigate dam contract issues, governance arrangements and economic and political implications, as well as their understanding of the social and environmental impacts of dams. For the community fieldwork, we carried out two FGDs in each of five affected villages around the Kamchay dam site in Cambodia – 10 FGDs in total. We also conducted 22 individual semi-structured interviews with community members and village chiefs. See also Siciliano et al. (2016) for parts of the fieldwork.

For the data analysis, we analyzed the transcribed, qualitative data by categorizing and coding the fieldwork sources as a means of comparing and contrasting interpretations of events (Wolcott, 1990). The interviews and FGDs were coded according to themes and sub-themes. The broad themes were divided into social, environmental, economic, political and technological perspectives (see also Siciliano et al., 2016).

## Findings: a comparative study of China’s dam-building in South-East Asia

### The role of Chinese actors in global dam-building

As the Asian Drivers approach highlights, analyzing the channels of interaction between China and dam host countries (such as aid, trade and investment) is crucial for understanding the complementary and competitive economic impacts, motives, actors and beneficiaries. This paragraph shows how Chinese investment strategies in large hydropower dams are managed vis-à-vis LMICs.

There are numerous Chinese dam-builders, and the companies registered and operating in China can vary from those subsidiaries specifically set up for overseas operation. These actors are usually the ones with the most power to inscribe change on the environment, and subsequently on livelihood options and power relations between the stakeholders at the local level. The main players in overseas hydropower dam-building are China Datang Overseas Investment Company (a subsidiary of Datang International Power Generation Company), Gezhouba International, China Huadian Corporation, China Huaneng Group, PowerChina Resources Limited (an international subsidiary of Sinohydro), Sinohydro Corporation (also known as PowerChina) and China Three Gorges Corporation. There are smaller players in the industry, such as suppliers and grid operators, including Hydro China, Dongfang, China Southern Grid and China State Grid. There are several Chinese dam financiers, such as the Export-Import Bank of China (ExIm Bank), Chinese Development Bank, Sinosure, and to a lesser extent commercial or non-policy banks, Industrial and Commercial Bank of China and the Bank of China (Tan-Mullins et al., forthcoming).

The Chinese government has been actively encouraging firms to invest overseas through its Going Out policy since 2000 (Mohan & Power, 2008). The government’s new One Belt, One Road project (Xinhua Finance Agency, 2015), a development strategy and framework initiated in 2013 to increase the economic and political ties among the former Silk Road countries, is also encouraging Chinese firms to operate overseas. The Mekong Region includes two of the six key One Belt corridors for expanded outward investment in infrastructure, namely the China-Indo-China Peninsula Economic Corridor and the Bangladesh-China-India-Myanmar Economic Corridor. One Belt, One Road qualifies Cambodia, Lao PDR, Myanmar, Thailand and Vietnam for a number of Chinese funding initiatives associated with infrastructure needs (Motta & Matthews, forthcoming). It is being reported that leading Chinese power firms have invested nearly US$3 billion overseas (FDI China, 2015).

Many of the companies’ respondents indicated that other than profits, the contributing factors for them to invest overseas include the Chinese government’s Going Out policy, stiff competition within China, sector reforms due to an increasingly saturated internal market, few suitable sites for new dams in China, lack of international competitors, and national policy directives. Low costs, access to finance (and at times cheap loans) and a big portfolio of domestic projects also make them attractive partners for clients around the world (Tan-Mullins et al., forthcoming):

Big dam-building is an important part of the Going Out strategy, but I think it is just business, it has no strong relationship with political issues. On the one hand, there is market demand; on the other hand, China has rich experience with dam-building. The domestic capacity in China is oversupplied, so Chinese dam firms need to go global. (Interview with ExIm Bank, 2013)

**Figure 1.**
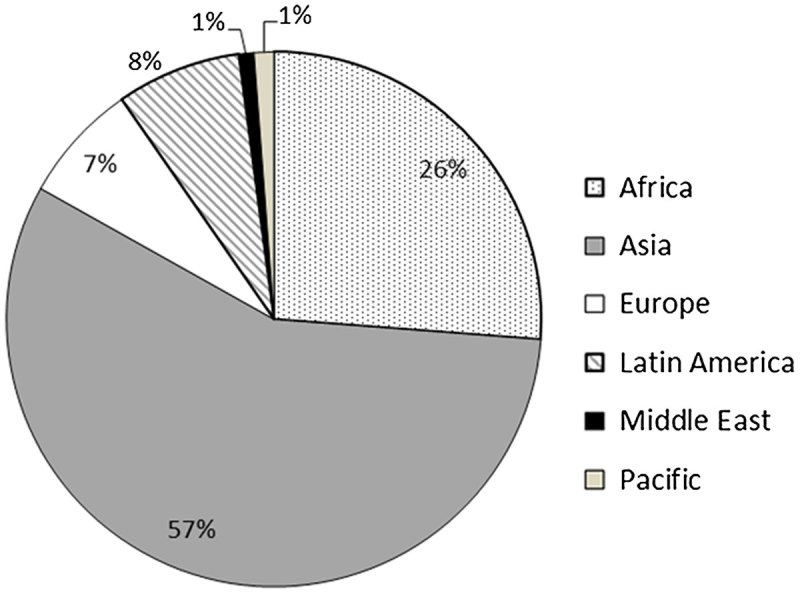
Chinese Overseas Dam Projects by Location – World Regions. Adapted from Urban and Siciliano (2013).

There are different Chinese ministries with varying degrees of involvement in the project cycle of an overseas Chinese dam project. They are the State Council, Ministry of Commerce (MOFCOM), Ministry of Foreign Affairs, National Development and Reform Commission (NDRC), Ministry of Environmental Protection, and the State-owned Assets Supervision and Administration Commission (SASAC) (Urban, Nordensvärd et al., 2013). Every project larger than US$2 billion requires approval from the State Council, endorsement by NDRC, and MOFCOM approval (International Rivers, November 2012, p. 7). The State Council is also the overarching agency that oversees MOFCOM and NDRC, two very important stakeholders in approving and overseeing overseas dam projects. MOFCOM is the main institution that approves and manages overseas investment by SOEs, which includes hydropower dams. NDRC approves smaller projects (less than US$2 billion) and regulates overseas investments. The Ministry of Foreign Affairs then provides advice on China’s foreign policy, such as aid matters, while the Ministry of Environmental Protection provides advice on environmental protection issues such as use of environmental impact assessments. Finally, SASAC assesses the performance of these SOEs (International Rivers, November 2012, pp. 8–9). As indicated above, although some decisions are driven more by national policies than by revenue, we should not overestimate the power of these ministries over the companies. The Chinese government can and does influence these companies if there is a strategic objective, but beyond this the companies are not generally under the control of the national government but are governed by boards and private investors (Tan-Mullins et al., forthcoming). Beneficiaries are primarily SOEs, but also the Chinese government, which receives tax income from the overseas operations. The following figures analyze China’s overseas dam investment. As Figure 1 indicates, the majority of the dams (whether completed, under construction or at the memorandum-of-understanding stage) are in Asia (57%, mainly in South-East Asia [38%]), followed by Africa (26%), Latin America (8%), Europe (7%, mainly Eastern Europe), and the Middle East and the Pacific (1% each), according to the International Rivers database (2015).

**Figure 2.**
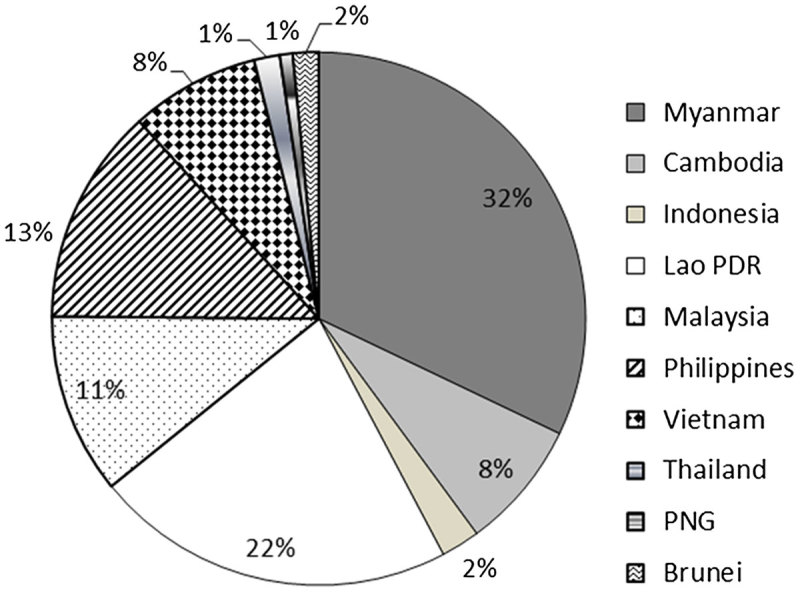
Chinese Overseas Dams in South-East Asia. Adapted from Urban & Siciliano (2013).

Looking at South-East Asia in particular, the most targeted countries are Myanmar, Laos, Philippines, Malaysia, Cambodia and Vietnam (Figure 2). Chinese dam activity in Vietnam has diminished in the last couple of years as the political tensions around the South China Sea and water scarcity on the downstream Mekong in Vietnam due to China’s dam-building activity upstream have worsened political relations between the two countries.

The Chinese are involved in overseas dam investments in different roles. They can act as financiers, developers, or builders, or as sub-contractors only, or a combination of these roles. Usually they are involved in at least two of the above tasks (Urban, Nordensvärd, et al., 2013).

**Figure 3.**
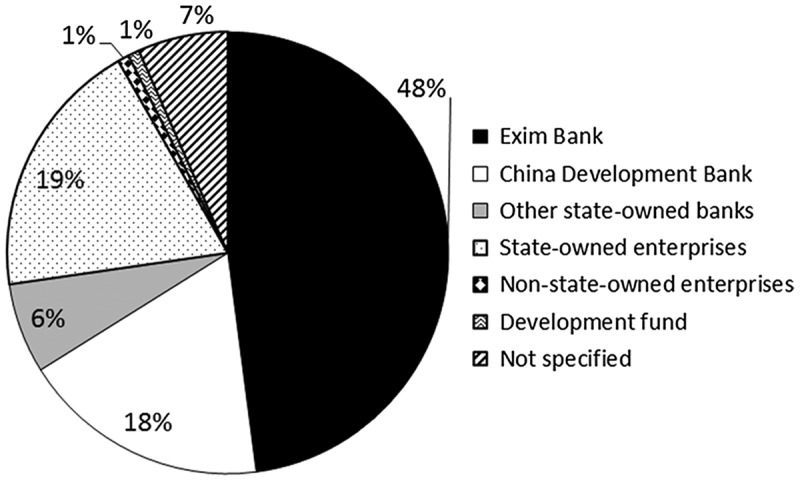
Chinese Financiers Involved in Overseas Dam Investments. Adapted from Urban and Siciliano (2013).

For the majority of dam investments (about 66%), China acts as the sole financier, according to the International Rivers database (2015). In 22% of the cases host national governments participate in the investment; for the remaining 13% cases international investors also participate together with Chinese dam-builders; and in few cases the investors are national financiers, such as the governments of host countries (Figure 3).

**Figure 4.**
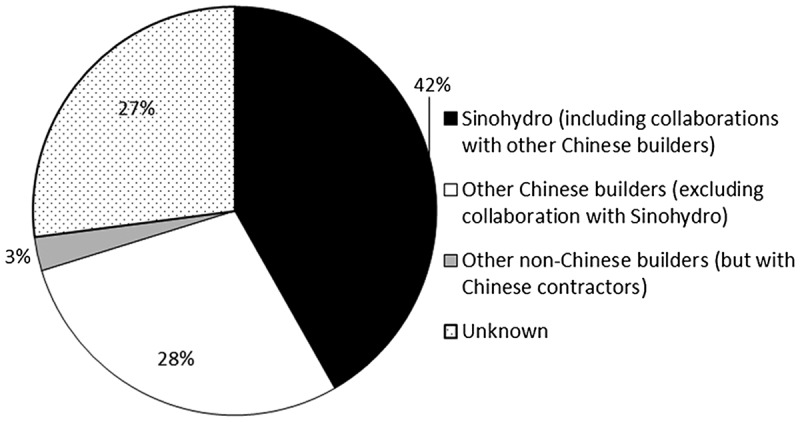
Chinese Builders Involved in Overseas Dam Projects. Adapted from Urban and Siciliano (2013).

Sinohydro leads the global hydropower sector as the world’s largest dam company in terms of number and size of dams built, investment amounts and global coverage (Figure 4). It is followed by China International Water & Electric Corp. and China Gezhouba Group Company Limited, both SOEs. Almost all of the Chinese companies involved in the construction of overseas dams are state-owned.

In the Greater Mekong Sub-Region, CGIAR’s WLE Greater Mekong Dam Database indicates that there are currently 87 dam developments led by Chinese overseas dam-builders, of which 57 are believed to be commissioned, 12 under construction, 16 planned or proposed, and 2 cancelled or suspended (CGIAR, 2016).

The next two sections deal with Chinese-built and Chinese-financed dams in Cambodia and Vietnam, respectively.

## Case study: Cambodia

### Cambodia’s energy setting

Cambodia had an electrification rate of only 31% in 2015, which means that almost 10 million people in Cambodia still lack energy access. The electrification rate in urban areas is high, at about 91%, but in rural areas it is very low, at only 19% (World Bank, 2016).

Electricity production in Cambodia relies mainly (90%) on oil products such as kerosene and diesel, with much smaller roles for hydro (4%), coal and peat (3%), biofuels (2%) and solar PV (0.3%). Fossil fuels are used for both transport and electricity generation. More than 90% of the electric supply comes from decentralized generators rather than the grid. Even the batteries that rural households use for lighting are charged at diesel-powered charging stations (Clean Energy Info Portal, 2013).

Cambodia’s electricity production is far below the country’s needs. Imports far exceed the internal electricity production, with roughly 1640 GWh of imports and 1050 GWh of internal production (IEA International Energy Agency, 2016). Cambodia therefore relies heavily on imported oil for the production of electricity, as well as on electricity imports, mainly from neighbouring countries, primarily Vietnam and Thailand. Thus, Cambodia has some of the highest electricity costs in the world, despite being a low-income country (IEA International Energy Agency, 2016). Electricity prices range from US$ 0.09–0.25 per kilowatt hour in urban areas connected to the central grid to US$ 0.40–0.80 per kilowatt hour in rural areas. For comparison, the average price in the USA is about US$0.12 per kilowatt hour (IEA International Energy Agency, 2016).

This creates difficulties in terms of improving energy access, especially in rural areas; imported electricity is very expensive, and that produced using fossil fuels depends greatly on the volatility of international fuel prices. As a consequence, the electrification rate is one of the lowest in Asia. Moreover, due to system inefficiencies and poor electrification networks in rural areas, rural households usually cannot afford to connect to the national power grid facilities. Cambodia also suffers from under-investment in grid infrastructure and power station infrastructure. The energy security situation in Cambodia is therefore rather unstable.

Poor energy access in rural areas is a critical aspect of Cambodia’s development also in terms of poverty reduction. According to World Bank data and estimates, in 2012, about 6% of the total population of Cambodia was below the poverty headcount of US$1.9 a day (PPP) and about 37% was below the poverty headcount of US$3.1 a day (PPP), corresponding to just under 5 million poor people (World Bank, 2014).

## Chinese investments in Cambodia’s hydropower sector

According to the National Strategic Development Plans for 2009–13 and 2014–18, energy, including from large hydropower, is central to sustainable growth and poverty reduction in Cambodia. Improving the power sector is one of the government’s key priorities for ensuring a reliable, secure electricity supply at affordable prices (Government of Cambodia, 2010).

Hydropower development is the highest energy priority in Cambodia; hydropower is expected to substantially replace fossil fuel consumption for electricity generation in the country. To increase the supply of electricity Cambodia plans to build and operate several new hydropower dams by 2019, supplying the country with 1942 MWe (Clean Energy Info Portal, 2013), including the dams already granted to Chinese companies by the Ministry of Industry, Mines and Energy of Cambodia, such as:

• Kamchay, 193 MW capacity (completed and operating)
• Stung Russey Chrum Krom, 338 MW (completed and operating)
• Stung Atai, 120 MW (completed and operating)
• Kirirom 1, 12 MW (completed and operating)
• Kirirom 3, 18 MW (completed and operating)
• Lower Sesan 2, 400 MW (under construction)
• Lower Sesam 3, 375 MW (under construction)
• Stung Tatai, 246 MW (under construction)
• Sambor, 7110 MW (proposed)
• Srepok 3, 300 MW (proposed)
• Srepok 4, 220 MW (proposed)
• Stung Cheay Areng, 108 MW (proposed).

In Cambodia no dams have been built directly on the mainstream Mekong River, but some are under way on tributaries. The Chinese dam-builders and financiers involved in these dams include both large and small players: financiers such as the ExIm Bank, lead firms such as Sinohydro and China Guodian, and smaller and less well-known firms such as Hydrolancang International, China Electric Power Technology Import and Export Corporation, and Guangxi Guiguan Electric Power (International Rivers, 2016).

Overall, the exploitable mid-to-long-term hydropower potential of Cambodia is estimated at 8600 MW by Chinese sources (Chinese Statistical Yearbooks, National Bureau of Statistics, 2011). The director of Kampot Provincial Department of Environment, which is responsible for the environmental management of the Kamchay dam, reported that the Kamchay dam alone could meet up to 60% of the country’s electricity demand in the wet season. However, in the dry season the capacity of the Kamchay dam falls to only a third, and this will only be exacerbated by climate change. In the long term, Cambodia hopes to export electricity to neighbouring countries to gain revenue. Cambodia’s Minister of Public Works and Transport, Khy Tainglim, sees the country’s future as follows: ‘Water is our oil … and we should use our water to export and get foreign currency to develop the country’ (quoted in Goh, 2004, p. 7).

The Cambodian commerce minister, Sun Chanthol, said, ‘Without infrastructure, you can’t revive’; and ‘we have been blamed for always going to China, but it is because we need infrastructure fast and quick, nothing more than that’ (SCMP, 2015, p. 1).

## Cambodia’s view on transboundary river management by China

Cambodia is likely to be impacted by Chinese-built dams in two different ways. One impact is likely to occur downstream as dams are built upstream on the Mekong River in neighbouring countries, particularly in Laos, such as the Xayabouri dam. Orr, Pittock, Chapagain, and Dumaresq (2012) suggest that building dams along the transboundary Mekong River is likely to reduce water flow, food security and nutritional intake for millions of people in the Greater Mekong Sub-Region. Cambodia is also affected by dams built within its own borders, such as the Lower Sesan II on the Mekong River, and dams built elsewhere, such as the Chinese-built and Chinese-financed Kamchay dam in Kampot Province, which was Cambodia’s first large-scale dam.

One could argue that China is attempting to move from the unilateral use of shared waters to joint developments and transboundary water cooperation. Yet, this view was not actively promoted by the interviewees, neither on the Chinese side nor on the Cambodian side. While Chinese dam-builders and financiers are investing heavily in Cambodia, their influence on Cambodia’s approach to transboundary water issues is not entirely clear. Many Cambodian interviewees did not raise major concerns that Chinese dam-builders may control water issues in Cambodia disproportionally.

However, with regard to specific dams and their influence, a lot depends on the type of contract between the dam-builders and the local government. Engineering, Procurement and Construction contracts are usually turn-key contracts, meaning that the dam-builders act merely as contractors, and hand over the ownership of the dam to local authorities after the construction is completed, so usually after a few years. Build-operate-transfer (BOT) contracts, on the other hand, mean that the ownership of the dam rests with the dam-building company for some decades, usually 20–40 years, before the dam is handed over to local authorities.

To give an example, the Kamchay dam is operating under a BOT contract with Sinohydro; the Chinese SOE will be in charge of the dam for 44 years (until 2050) before ownership is passed on to the Cambodian authorities. The revenues from selling the electricity also accrue to Sinohydro during this time, while the management of the dam remains firmly in Chinese hands for four decades. This means that Cambodian authorities have limited opportunity to operate the dam and control its impacts, even though the dam is in Cambodia. This speaks against the idea of China’s moving from the unilateral use of shared waters to joint developments and transboundary water cooperation and reinforces the view that Chinese firms will be able to control water issues in the Greater Mekong Sub-Region for several decades if more dams go ahead with BOT contracts. Interestingly, the lengthy time scale of BOT contracts did not seem to worry many Cambodian interviewees. It did however worry international NGOs operating in Cambodia, as well as researchers.

At the same time, Cambodia has very limited negotiation and decision-making power with regard to China’s development activities on the upstream of the Mekong River basin. Having more Chinese-funded and Chinese-built dams in Cambodia does not seem to increase the leverage or power Cambodia has over upstream water issues.

## Political Ecology of the Asian Drivers: Cambodia

As indicated in the conceptual framework section, we focus on the following channels of interaction: trade, investment, aid, innovation, politics, environment and society. We also analyze the motives, actors, beneficiaries and impacts from both the Chinese side and the LMIC side.

With regard to the *motives* for Chinese dam-building in Cambodia, our research finds the following. China is one of Cambodia’s largest trading partners. China is also one of Cambodia’s largest investment partners, and of strategic importance as Western countries don’t invest much in Cambodia. In the dam sector, Chinese actors bundle aid, trade and investment. This enables lucrative trade deals for Chinese firms. The Cambodian hydropower market is underdeveloped, and there is limited domestic expertise; hence Chinese dam-builders can invest in and exploit a largely untapped market, making profits, providing jobs and generating tax income for China and the Chinese. With regard to aid, China is a big donor in Cambodia and mainly invests in infrastructure such as roads, bridges and dams. China provides a no-strings-attached, non-conditionality aid opportunity that rivals Western donors. ‘No strings attached’ is a term used in development studies and international relations to describe China’s approach to aid, trade and investment projects in the global South. Chinese actors do not attach any conditionality to their projects, as Western donors such as the World Bank do; they do not get involved in the internal politics of a country. They therefore also invest in countries where, for political reasons, Western donors seldom do, such as Sudan and North Korea. With regard to innovation, Chinese dams are state-of-the-art and enable Cambodia to access the latest hydropower technology at a reasonable cost. In return for political and economic support from China, Cambodia is supportive of China’s One China policy. Cambodia also sides with China regarding the South China Sea dispute, in exchange for aid, trade and investment deals. With regard to environmental motives, hydropower dams contribute to low-carbon energy generation and may deliver cost-effective electricity in the long term. Societal motives include bringing progress, modernity and electricity to an underdeveloped country.

For aid, trade and investments, *actors* include Chinese dam-builders – hydropower firms as well as financiers – and other firms along the hydropower value chain, as well as traders securing deals for other goods, and Chinese and Cambodian authorities and ministries. With regard to innovation, actors include Chinese dam-builders, with Cambodian firms and the state being the recipients of dam innovation in the long run. However, ownership transfer can be delayed several decades due to BOT contracts. With regard to politics, the actors are Chinese and Cambodian authorities and ministries. With regard to the environment, the actors are Chinese dam-builders, as well as Cambodian authorities that sign off on environmental impact assessments and grant licences. Actors in society include local communities, mainly in rural areas and often impoverished, who are usually affected the hardest as the dam affects them and their livelihoods directly. Urban areas and industries tend to benefit from the delivery of electricity.

For aid, trade and investment, the *beneficiaries* are the dam-builders – Chinese firms and financiers – and the Cambodian state and people, as well as Cambodian firms that cooperate on trade and investment deals with China. For politics, the beneficiaries are Chinese and Cambodian authorities. For the environment, the main beneficiary is the global atmosphere, as the hydropower dams can replace fossil fuel–powered stations and thereby contribute to climate change mitigation. In terms of society, the beneficiaries are predominantly people living in urban areas, industries and services that receive electricity, dam-builders, financiers and the political elite.

The *impacts* are both positive and negative, as well as both direct and indirect. Direct positive trade, investment and aid impacts mean increased trade, investment and aid relationships between China and Cambodia, mainly based on trade, imports and aid from China to Cambodia. Indirect positive impacts are potential export opportunities from Cambodian firms to China, although on a small scale. Negative impacts are trade, investment and aid dependencies on China. An indirect negative impact is Cambodia potentially becoming unattractive for Western donors due to the Chinese dominance. For innovation, the direct positive impacts are access to state-of-the-art dam technology for Cambodia, while an indirect positive impact is knowledge transfer from Chinese actors to local Cambodian firms and engineers. Direct negative impacts relate to BOT contracts’ meaning that innovation and knowledge will be transferred from Chinese dam-builders to Cambodian actors only after several decades, rather than after a few years or immediately after construction. With regard to politics, direct positive impacts are support for China on a domestic and regional basis and China’s political support for Cambodia. Indirect positive impacts are stronger bilateral ties, and an indirect negative impact is the potential for conflict between Cambodia and other countries that do not support the One China policy or China’s claims in the South China Sea. In terms of the environment, the major direct positive impact is the reduction of greenhouse gas emissions from energy generation. Direct negative impacts are local and transboundary impacts, including changes in hydrology, geomorphology, impacts on fish stocks, water quality and quantity, loss of habitat for fauna and flora, and erosion. Indirect negative impacts are impacts on food security for millions of people along the Mekong River. With regard to society, direct positive impacts include access to electricity. Electricity is provided mainly for urban areas, particularly Phnom Penh. Direct negative impacts are local and transboundary impacts, such as declines in or even loss of livelihoods, resettlement, sometimes inadequate compensation issues, and potential impacts on food security. Indirect negative impacts relate to transboundary impacts on people and communities downstream.

## Case study: Vietnam

### Vietnam’s energy setting

Vietnam had an electrification rate of 99% in 2015 – 100% in urban areas and 97% in rural areas (World Bank, 2016). The country is therefore far less dependent on building dams or other new power generation infrastructure than some of its neighbouring countries. About 8% of the primary energy supply and 40% of electricity supply come from hydropower, but the country also depends heavily on fossil fuels: coal, natural gas and oil (IEA International Energy Agency, 2016).

### Chinese investments in Vietnam’s hydropower sector

China and Vietnam share a long and complex history of political, economic and social ties (Rigg, 2003). According to Lamb and Dao (2015), China is Vietnam’s largest trading partner today. Middleton suggests that energy trade accounts for about 20% of bilateral trade between China and Vietnam. Vietnam exports coal to China, while China exports electricity to Vietnam (Middleton, 2008). China is involved in the electricity sector by supplying a small share of Vietnam’s electricity supply, which is imported from China at three times the price of the local Vietnamese electricity (Lamb & Dao, 2015). Not purchasing the electricity from China carries a penalty, so the electricity is imported even when there is no demand for it (Lamb & Dao, 2015).

Vietnam has between 12,000 MW (Chinese Statistical Yearbooks, National Bureau of Statistics, 2011) and 17,000 MW (Middleton, 2008) of economically and technically exploitable hydropower resources. Chinese dam-builders and financiers have been involved in the construction of four dams in Vietnam, at Cua Dat, Nam Mu, Tuyen Quang and Zhan Hua, in addition to several others planned or under construction (International Rivers, 2016). These hydropower projects include the involvement of Chinese firms such as China National Heavy Machinery Corporation, Dong Fang Electrical Machinery Company, Yunnan Machinery Export Import Company, and Guangdong No. 2 Hydropower Engineering Co. Ltd. The completed projects did not involve major lead firms such as Sinohydro or China Three Georges Corporation (International Rivers, 2016).

While China’s involvement in the Vietnamese hydropower sector may appear minor in comparison to other countries in the Greater Mekong Sub-Region such as Cambodia, Laos and Myanmar, China also supplies hydropower equipment and workers and is involved as a business partner in larger dam contracts led by Vietnam (Lamb & Dao, 2015). Vietnam is itself a leader in dam-building, so its hydropower firms compete for contracts with Chinese firms. Many of its hydropower projects are constructed and/or owned by Electricity of Vietnam, a large utility SOE. Yet, Chinese dam-builders are often involved in project design, construction, and/or equipment provision. Lamb and Dao (2015) argue that up to 90% of Vietnamese dams are supplied with equipment by Chinese dam-builders. Also, Chinese workers are often used for dam projects in Vietnam. Many of the dams in which Chinese actors are involved are small-scale (below 30 MW), so reliable data are more difficult to obtain. An example is the Coc San hydropower project, for which China Southern Power Grid Company partnered with Electricity of Vietnam to develop a 21.4 MW hydropower station in Lao Cai Province, with an estimated value of US$28 million (Middleton, 2008). When it comes to water management and security, Vietnamese authorities generally have a rather negative perception and limited trust of Chinese infrastructure operations, especially in relation to dam safety and security systems. According to an engineer in charge of the operation of the Coc San dam interviewed in October 2016, while responsibility for construction and installation of hydropower dams in Vietnam can be given to Chinese companies, the safety and security systems usually come from European or American companies, which are perceived as more serious in terms of monitoring the state of dams and their vulnerability to external physical threats and risks, such as landslides.

## Vietnam’s view on transboundary river management by China

### Governance for transboundary river management

Cross-border cooperation on hydropower and water management between China, Cambodia, Vietnam and the other countries located upstream, such as Myanmar, Laos and Thailand, is essential to avoid severe impacts on food security and the environment of the Mekong Delta region. Orr et al. (2012) suggest that building dams along the transboundary Mekong River is likely to reduce water flow, fish stocks and hence fish catch, which may reduce food security and nutritional intake for millions of people in the Greater Mekong Sub-Region. With regard to transboundary river management, the Mekong River Commission (MRC) oversees the management of the Mekong River, but it does not have enforcing power. Before dams are built on the river, consultation of the MRC and member states needs to happen, but the concerns of neighbouring countries, scientists and environmentalists are often neglected. Sometimes, such as with the Xayaburi dam in Laos, construction has started before the consultation process was completed (Zaffos, 2014). The MRC has even less power when dams are built on tributaries of the Mekong River. The MRC works directly with the governments of Cambodia, Laos, Thailand and Vietnam on transboundary river management. However, there is no legal requirement to inform or consult other countries along the Mekong River before building dams, and China, as a non-member of the MRC, may be even less inclined to inform or consult other countries along the Mekong River. However, the new Lancang-Mekong Cooperation Mechanism (established in November 2015), which includes cooperation on water resources, could play a fundamental role in increasing transparency and communication between China and the other Mekong countries. The new mechanism covers five different priority areas: interconnectivity; production capacity; cross-border economic cooperation; water resources; and cooperation on agriculture and poverty reduction. With this mechanism China may be aiming to engage in a more active and positive role in water resources management in the Mekong region by increasing its cooperation and transparency of operations. This could in turn build trust between Chinese investors and the other Mekong countries in relation to hydropower development and water management (Biba, 2016).

### Domestic views from Vietnam on transboundary river management

While Cambodia depends on and welcomes Chinese investments in the hydropower sector to enable economic growth and modernization, the situation is different in Vietnam. Unlike Cambodia, Vietnam is located all the way downstream, at the very end of the Mekong River where it pours into the sea. Any dams built upstream on the Mekong River in China, Cambodia, Laos, Myanmar or Thailand could significantly reduce water levels in Vietnam and cause water scarcity and a threat to food security. Vietnam’s location downstream makes it very vulnerable to declines in water levels and fish stocks; it is disproportionally affected by dam-building upstream. Zaffos (2014, p. 1) writes:

Seven dams built upstream in China and the blasting of rapids to improve navigation have already altered flows, reduced fish populations, and affected communities along portions of the Lower Mekong, which flows through Thailand, Laos, Cambodia, and Vietnam. But the impacts may soon get much worse as a new era of hydroelectric dam-building begins in the Lower Mekong Basin. Eleven major hydroelectric dams – mostly within Laos – and dozens of dams on tributary streams that feed into the Mekong have been proposed or are under construction.

About 50 million people depend on the Mekong – the rice bowl of Asia – for their livelihoods and food security (Urban, Nordensvärd, et al., 2013). Orr et al. (2012) suggest that food security is at a high risk if more dams are built in the Lower Mekong River Basin as fish catches are likely to be reduced by declining fish stocks.

The Vietnamese government is also concerned about the impacts of new hydropower dams on the upper reaches of the Mekong River in Cambodia, which could affect water quantity in Cambodia’s Tonle Sap Lake. The deputy minister of agriculture and rural development of Vietnam is particularly concerned that if water flow decreases due to hydropower development in Cambodia, the Cambodian government may divert water from the Tonle Sap Lake for agricultural production. This could reduce water availability in Vietnam, since the Mekong River reaches the Vietnamese Mekong Delta region through the Tonle Sap Lake (VietNamNet Bridge, 2016). Scientific studies have also demonstrated that upstream water infrastructure on the Mekong River in Cambodia and other neighbour countries, such as Laos, has changed the water flood and water levels of the Tonle Sap Lake (Cochrane, Arias, & Piman, 2014). Moreover, Vietnamese authorities are concerned that hydropower dams upstream could result in the Mekong River Delta in Vietnam in either floods, due to the sudden and uncontrolled release of water from upstream dams, or drought, due to the use of water for hydropower or irrigation purposes in upstream countries (Soksreinith, 2016; VietNamNet Bridge, 2016).

In 2015–16, Vietnam was very badly affected by climatic changes, manifested particularly in a record drought exacerbated by El Niño. Government officials argue that the drought is being further exacerbated by Chinese dams on the Upper Mekong River, and they are concerned about the impacts of the current dam-building on the Lower Mekong River (interview with Vietnamese government official, 2016). A recent study by Vietnam for the MRC indicates that further dam construction could have devastating impacts on Vietnam and also Cambodia.

The dam-building and its severe downstream impacts have caused concern among government officials, experts and scholars, some arguing that the Mekong River could soon be considered a diplomatic challenge akin to the South China Sea, which could potentially threaten Sino–ASEAN relations. Bilateral relations between China and Vietnam have certainly turned sour. A senior Vietnamese government official interviewed in 2016 put it this way:

The relationship between Vietnam and China has always been difficult. Several years ago, we were positive about Chinese engagement with Vietnam, including about bilateral trade and Chinese investments in Vietnam. But this has changed dramatically. The problems in the South China Sea and the dams that China has built and is building on the Mekong River are serious challenges for us. Also, Vietnam is experiencing a terrible drought this year. In the Mekong Delta, the water is scarce due to the dams upstream. In other areas of the country, farmers are advised to dig wells to find water, but there is no groundwater, not even after digging boreholes 150 metres deep.

Upstream dam-building and the exacerbative impacts of climate change are likely to put additional strain on already tense relations between China and Vietnam.

Like Cambodia, Vietnam has very limited negotiation and decision-making powers with regard to China’s development activities on the upstream of the Mekong River basin. Having a few Chinese-funded and Chinese-built dams in Vietnam does not seem to increase the leverage or power the country has over upstream water issues. Yet, an improved way for both countries to negotiate transboundary water management issues with China is through the Lancang-Mekong Cooperation Mechanism.

## Political Ecology of the Asian Drivers: Vietnam

As indicated in the conceptual framework section, we focus on the following channels of interaction: trade, investment, aid, innovation, politics, environment and society. We also analyze the motives, actors, beneficiaries and impacts from both the Chinese side and the LMIC side.

With regard to the *motives* for Chinese dam-building in Vietnam, our research finds the following. China is Vietnam’s largest trading partner and one of Vietnam’s largest investment partners. There is less evidence of the bundling of aid, trade and investment in the dam sector by Chinese actors in Vietnam. The Vietnamese hydropower market is well developed, with experienced domestic firms and domestic expertise; hence Chinese dam-builders play only a minor role alongside Vietnamese dam-builders, often along the value chain in terms of planning and contracting. With regard to aid, as Vietnam is a middle-income country, it receives less aid from China than other Asian countries do. With regard to innovation, Chinese dams are state-of-the-art; yet often Vietnamese dams are built by Vietnamese dam-builders and Chinese dam-builders play a minor role as contractors for specific parts of the dam-building. There is less evidence of transfer of innovation. Political motives are complex. Vietnam is supportive of China’s One China policy, in return for political and economic support from China. Yet, Vietnam’s political ties with China have historically been difficult and are now being strained by extensive dam-building upstream on the Mekong River, which could harm Vietnam’s food security and water availability. Vietnam is also claiming parts of the South China Sea for itself, hence opposing China on this matter. With regard to environmental motives, hydropower dams contribute to low-carbon energy generation, contribute to climate change mitigation, and may deliver cost-effective electricity in the long term. Societal motives include bringing progress, modernity and electricity to Vietnam.

For aid, trade and investments, *actors* include Chinese dam-builders – hydropower firms as well as financiers – often operating alongside Vietnamese firms; other firms along the hydropower value chain; traders securing deals for other goods; and Chinese authorities and ministries. With regard to innovation, actors include Vietnamese and Chinese dam-builders. With regard to politics, the actors are Chinese and Vietnamese authorities and ministries. Vietnamese authorities are concerned regarding upstream dam-building by China on the Mekong River. With regard to the environment, the actors are Chinese dam-builders, as well as Vietnamese authorities that sign off on environmental impact assessments and grant licences. With regard to society, the actors are local communities downstream, particularly in the Vietnamese Mekong Delta. These are mainly in rural areas and sometimes impoverished; livelihoods are directly affected. Some benefit from electricity access.

For aid, trade and investment, the *beneficiaries* are dam-builders, Chinese firms and Vietnamese firms. For politics, the beneficiaries are Chinese and Vietnamese authorities. For the environment, the main beneficiary is the global atmosphere, as hydropower dams can replace fossil fuel–powered stations. In terms of society, the beneficiaries are predominantly people living in urban areas, industries and services receiving electricity, dam-builders, financiers and the political elite.

The *impacts* are both positive and negative, as well as both direct and indirect. Direct positive trade, investment and aid impacts mean increased trade, investment and, to a small extent, aid relationships between China and Vietnam. There are opportunities along the dam value chain for Chinese firms in Vietnam. Indirect positive impacts are potential export opportunities from Vietnamese firms to China. Negative impacts are trade and investment dependencies on China. For innovation, the direct positive impacts are know-how and expertise for state-of-the-art dam technology for Vietnam, although direct knowledge transfer to local firms and engineers seems limited. With regard to politics, a direct positive impact is the partial political support for China on a domestic and regional basis. Direct negative impacts include political tension due to a series of dams built upstream on the Mekong River. Indirect negative impacts include the potential for conflict with countries that support China unconditionally. In terms of the environment, the major direct positive impact is the reduction of greenhouse gas emissions from energy generation. Direct negative impacts are changes in hydrology and geomorphology and impacts on fish stocks, water quality and quantity, habitat for fauna and flora, and erosion. Local and transboundary impacts are most important for upstream dams in China, Lao PDR and Cambodia that impact the Mekong Delta downstream in Vietnam. Indirect negative impacts include impacts on food security for millions of people along the Mekong River and especially in the Mekong Delta. With regard to society, direct positive impacts include access to electricity. Direct negative impacts are decline in or even loss of livelihoods, resettlement, sometimes inadequate compensation issues, and sometimes impacts on food security. There is a range of local and transboundary impacts, where the most severe impacts are expected in the Mekong Delta due to dams built upstream by Chinese dam-builders. Indirect negative impacts relate to food security threats in areas that depend on Vietnam’s food production, particularly rice production, and this may even affect countries that import rice and other agricultural produce from Vietnam.

## Discussion and conclusion

The analysis presented above finds that China’s dam-building activities are perceived very differently by the interviewees in different countries of South-East Asia, depending on whether they can be helpful for economic growth and development in a poor country such as Cambodia, or whether they threaten growth and development, as well as national security, in a downstream, better-off country such as Vietnam.

Transboundary water management along the Mekong River has become a serious challenge in recent years. China’s building of large dams upstream within its own borders on the Lancang/Upper Mekong and in Cambodia and Laos on the Mekong River means that relations between downstream Vietnam and China are strained. Across the ASEAN region there is concern that the excessive damming of the Mekong and its tributaries by Chinese dam-builders and others may lead to water scarcity and food insecurity across the region.

As China is not a part of ASEAN, it is difficult to hold the country accountable and to bring it to the negotiating table to discuss transboundary river management and international water governance. However, the newly established Lancang-Mekong Cooperation Mechanism, which includes cooperation on water resources, could play a fundamental role in increasing transparency and communication between China and the other Mekong countries. Yet, the media and some critics accuse China of dealing with the Lancang/Upper Mekong as if it were only a domestic river, largely ignoring the downstream impacts. At the same time, the country’s dam-builders do not seem very concerned about potential transboundary and regional problems caused by the dams, such as water scarcity and threats to food security. There is also a lack of data, meaning that few transboundary studies exist yet to quantify the potential impacts of these large dams on fish stocks, food security and water security (Orr et al., 2012, is an outstanding exception). More research in this area is needed.

Some scholars (e.g. Guo, 2016; Zhang, 2014; Zhang & Lu, 2015) argue that China is attempting to move from the unilateral use of shared waters to joint developments and transboundary water cooperation. Yet, this view was not actively promoted by the interviewees, whether on the Chinese side, the Cambodian side, or the Vietnamese side. While Chinese dam-builders and financiers invest in Cambodia and Vietnam, the influence they have on transboundary water management depends partly on the types of contracts being used. Engineering, Procurement and Construction contracts mean that Chinese firms and financiers have very limited influence over a particular dam (or a series of dams) over time. BOT contracts mean that the ownership of the dam rests with the dam-building company for some decades (up to 40 years) before the dam is handed over to local authorities. This reduces the power local authorities have to operate the dam and control its impacts, even though the dam is located within their national boundaries. This speaks against the idea that China is moving towards joint developments and transboundary water cooperation and reinforces the view that Chinese firms will be able to control water issues in the Greater Mekong Sub-Region for several decades if more dams go ahead with BOT contracts. Yet, this does not seem to be the motivation of the Chinese government. Instead, the main reasons for China’s dam industry to go global are economic and only partly political (Matthews & Motta, 2013; Urban, Nordensvärd, et al., 2013).

At the same time, Cambodia and Vietnam have very limited negotiation power and decision-making power with regard to China’s development activities on the upstream of the Mekong River basin. And having more Chinese-funded and Chinese-built dams in these countries does not seem to increase the leverage these countries have over upstream water issues, despite the closer economic and political ties they bring with China.

Using the conceptual framework of the Political Ecology of the Asian Drivers, the article finds that different channels of interaction are used in the dam sector to engage between Chinese dam-builders and financiers and host countries. In Cambodia, the bundling of aid, trade and investments in the dam sector is far more common than it is in Vietnam. Compared to Cambodia, Vietnam has relatively little investment from China in the dam sector, and bilateral hostilities around the South China Sea have not improved this situation. With regard to political ecology considerations, the following assumptions have been confirmed. First, costs and benefits associated with environmental change are distributed unequally. Second, this unequal distribution inevitably reinforces or reduces existing social and economic inequalities. Third, the unequal distribution of costs and benefits and the reinforcing or reducing of pre-existing inequalities have political implications in terms of altered power relationships. The costs and benefits of large dam-building are indeed unequally distributed. Poor people are disproportionally affected by large dams, such as people depending on fisheries and the ‘rice bowl of Asia’ irrigated by the Mekong River. The winners are Chinese firms, mostly SOEs, as well as local elites such as in the Cambodian government. This exacerbates existing inequalities and affects the poor disproportionally. Finally, the costs and benefits of large dam-building in South-East Asia have political implications, as Chinese investors are welcome alternatives to the OECD donors, at least upstream, who have been preaching conditionality and structural reforms for decades.

This project finds that Chinese dam-builders and financiers open up opportunities for LMICs in Asia to attract large investments, to build up energy and water management infrastructure, which in turn can contribute to national development goals. Accessibility of finance and technical expertise from China, without the conditionality that might be uncomfortable for the local political economy, facilitates transformational and environmentally and socially high-risk projects that are otherwise limited by the conditionality and practice of agencies such as the World Bank and by the higher opportunity cost of other sources of finance. The no-strings-attached policy of Chinese dam-builders is a welcome offer to many LMICs. Hydropower dams also contribute to low-carbon energy generation and thereby create viable alternatives to fossil fuel energy generation, such as coal, oil and natural gas, thereby mitigating climate change. However, dam planning and building need to be done in a more sustainable way that takes into account national development priorities, the needs of local people, impacts on natural habitats, and cross-border impacts. To be more specific in outlining the implications for global hydropower development, this will require more cooperation across various countries to enable valuable negotiations on transboundary river management and international water governance. As rivers know no boundaries, countries need to work more closely together to deal with the impacts of planned dams on transboundary rivers such as the Mekong, to raise concerns beyond their own borders, and if possible to get a mandate to veto controversial dam projects that could be detrimental to them or neighbouring countries.

With regard to the role of Chinese dam-builders, our research finds that indeed the corporate behaviour of Chinese dam-builders is to a large extent influenced by legislation, policies and practices set by the national governments in Asia, but international institutions and industry bodies, such as the World Bank and the International Hydropower Association, do further provide international standards and monitoring for responsible corporate behaviour in the Chinese hydropower sector. In terms of policy recommendations, though, this means that Chinese dam-builders need to be willing to take on the recommendations of the World Bank and the International Hydropower Association, which is today rather limited practice. Countries on the downstream of transboundary rivers, such as Cambodia and Vietnam, need a much stronger opportunity to voice their concerns and raise suggestions for how to work with Chinese dam-builders and financiers. By working together, showing more willingness to improve the hydropower sector, and showing more consideration for transboundary water problems, Chinese dam-builders and financiers, national host governments, and international public institutions and regulatory bodies such as the MRC and the Lancang-Mekong Cooperation Mechanism could help make the hydropower sector more sustainable and reduce negative impacts on local people and the environment. It also needs to be considered whether large dams are appropriate for certain settings, or whether several smaller hydropower schemes or other forms of renewable energy would be more suitable to cater to the development needs of LMICs.

## Disclosure statement

No potential conflict of interest was reported by the authors.

## Funding

This work was supported by the UK Economic and Social Research Council [ES/J01320X/1], the Volkswagen Foundation, the Wellcome Trust and the Svenska Riksbankens Jubileumsfond.
